# Complete Genome Sequence of *Torque teno indri virus 1*, a Novel Anellovirus in Blood from a Free-Living Lemur

**DOI:** 10.1128/genomeA.00698-17

**Published:** 2017-07-27

**Authors:** Rina Amatya, Sharon L. Deem, Ingrid J. Porton, David Wang, Efrem S. Lim

**Affiliations:** a Departments of Molecular Microbiology and Pathology & Immunology, Washington University School of Medicine, St. Louis, Missouri, USA; b Institute for Conservation Medicine, Saint Louis Zoo, St. Louis, Missouri, USA; c Madagascar Fauna and Flora Group, St. Louis, Missouri, USA; d School of Life Sciences, Arizona State University, Tempe, Arizona, USA; e Center for Fundamental and Applied Microbiomics, The Biodesign Institute, Arizona State University, Tempe, Arizona, USA

## Abstract

We identified *Torque teno indri virus 1* (TTIV1), the first anellovirus in a free-living lemur (*Indri indri*). The complete circular 2,572-nucleotide (nt) TTIV1 genome is distantly related to torque teno sus virus. Phylogenetic and sequence analyses support TTIV1 as a putative member of a new genus within the *Anelloviridae* family.

## GENOME ANNOUNCEMENT

Anelloviruses, also commonly called torque teno viruses, are nonenveloped viruses that encode a circular single-stranded DNA genome. While members of the *Anelloviridae* family have high genetic variability, they share a similar genome organization with three to four open reading frames (ORFs), conserved sequence motifs, and a conserved noncoding GC-rich region (1). There are currently 12 genera within the *Anelloviridae* family that are found in a host-species-specific manner. For example, human anelloviruses include torque teno virus (*Alphatorquevirus* genus), torque teno minivirus (*Betatorquevirus*), and torque teno midi virus (*Gammatorquevirus*) (2, 3). In addition, torque teno sus virus (*Iotatorquevirus*) and torque teno sus virus k2 (*Kappatorquevirus*) are found in swine (4, 5). Other animal hosts include a wide range of species, including cats, gorillas, chimpanzees, and chickens (6–8). While anelloviruses are frequently detected in blood specimens, they have not yet been associated with specific pathology or disease (9).

As part of ongoing efforts to characterize novel viruses (10), we performed unbiased metagenomic next-generation sequencing (Illumina MiSeq 2x 250 v2, Illumina, San Diego, CA, USA) of a blood specimen collected from a free-living lemur (*Indri indri*) in the Betampona Nature Reserve, Toamasina Province, Madagascar (S17.931389 and E49.20333). Bioinformatic analyses performed using the VirusSeeker computational pipeline (11) identified Illumina sequencing reads with limited identity to known anelloviruses. The resulting complete genome was amplified in overlapping PCR fragments using high-fidelity DNA polymerase (Accuprime Pfx DNA polymerase [Invitrogen, Carlsbad, CA, USA]), cloned, and Sanger sequenced to 3× genome coverage as previously described (12). This virus was named *Torque teno indri virus 1* (TTIV1).

The complete circular genome of TTIV1 was 2,572 nucleotides (nt) in length with three predicted partially overlapping ORFs, including the 520-amino-acid (aa) ORF1, which encodes the putative capsid protein (13). Consistent with other previously described anellovirus genomes (14), the TTIV1 genome has a short (<50 nt) GC-rich sequence (nt position 40 to 86, 83% GC content) in the noncoding region, as well as a conserved WX_7_HX_3_CXCX_5_H motif within ORF2. Phylogenetic analyses of ORF1 amino acid sequences from representative members of the *Anelloviridae* family (*n* = 91) demonstrated that TTIV1 is a distinct lineage that is distantly related to torque teno sus virus, its closest relative. The International Committee on Taxonomy of Viruses (ICTV) guidelines for anellovirus genera and species are based on >56% and >35% cutoff values, respectively, in nt sequence divergence of the entire ORF1 (15). The highest nt sequence identity of TTIV1 ORF1 to torque teno sus virus ORF1 sequences (*n* = 47) was 39.63% (i.e., 60.37% sequence divergence to torque teno sus virus 1b isolate [GenBank accession number JX535332]). Thus, given the unique lemur host, phylogenetic relationship, and high sequence divergence to known anelloviruses, TTIV1 should be classified as a member of a novel genus in the *Anelloviridae* family. This is the first reported complete DNA virus genome from lemurs, and this study supports the potential for unique viral diversity in lemurs.

### Accession number(s).

The complete TTIV1 genome sequence has been deposited in GenBank under the accession number MF187212.
